# Conservation of context-dependent splicing activity in distant Muscleblind homologs

**DOI:** 10.1093/nar/gkw735

**Published:** 2016-08-23

**Authors:** Julia C. Oddo, Tanvi Saxena, Ona L. McConnell, J. Andrew Berglund, Eric T. Wang

**Affiliations:** 1 Koch Institute for Integrative Cancer Research, Massachusetts Institute of Technology, Cambridge, MA 02139, USA; 2 Department of Molecular Genetics and Microbiology, University of Florida, Gainesville, FL 32610, USA; 3 Center for Neurogenetics, University of Florida, Gainesville, FL 32610, USA; 4 Department of Biochemistry and Molecular Biology, University of Florida, Gainesville, FL 32610, USA; 5 Department of Chemistry and Biochemistry and Institute of Molecular Biology, University of Oregon, Eugene, OR 97403, USA

## Abstract

The Muscleblind (MBL) protein family is a deeply conserved family of RNA binding proteins that regulate alternative splicing, alternative polyadenylation, RNA stability and RNA localization. Their inactivation due to sequestration by expanded CUG repeats causes symptoms in the neuromuscular disease myotonic dystrophy. MBL zinc fingers are the most highly conserved portion of these proteins, and directly interact with RNA. We identified putative MBL homologs in *Ciona intestinalis* and *Trichoplax adhaerens*, and investigated their ability, as well as that of MBL homologs from human/mouse, fly and worm, to regulate alternative splicing. We found that all homologs can regulate alternative splicing in mouse cells, with some regulating over 100 events. The *cis*-elements through which each homolog exerts its splicing activities are likely to be highly similar to mammalian Muscleblind-like proteins (MBNLs), as suggested by motif analyses and the ability of expanded CUG repeats to inactivate homolog-mediated splicing. While regulation of specific target exons by MBL/MBNL has not been broadly conserved across these species, genes enriched for MBL/MBNL binding sites in their introns may play roles in cell adhesion, ion transport and axon guidance, among other biological pathways, suggesting a specific, conserved role for these proteins across a broad range of metazoan species.

## INTRODUCTION

Splicing is a co- or post-transcriptional process in which the spliceosome catalyzes excision of introns, or non-coding regions, from a precursor RNA transcript while concomitantly joining exons, or coding regions. This structure of exons and introns enables the generation of multiple mRNA isoforms from a single gene through the exclusion and inclusion of exons and introns, leading to multiple protein isoforms. Alternative splicing is a process that contributes to the vast diversity observed in fungi, plant and animal proteomes. *Trans*-acting protein factors can function as regulators of alternative splicing by interacting with specific sequences, or RNA secondary structures, termed splicing regulatory elements, within the RNA transcript to regulate spliceosome recruitment to or interaction with splice sites. These regulators can act in a spatio-temporal and developmentally dependent manner to enhance or repress inclusion of alternative exons (1,2).

Muscleblind (MBL) is a conserved family of RNA-interacting proteins that regulate many aspects of RNA metabolism, including tissue- and development-specific activation or repression of alternative splicing (3). Plants, fungi and bacteria lack any protein that resembles MBL, so it appears that this family is exclusive to metazoans, evolving approximately 800 million years ago. Typically, invertebrates encode a single *MBL* gene, whereas vertebrates encode multiple *MBL* genes; humans and other mammals have three muscleblind-like (*MBNL1-3*) genes. For simplicity we will refer to all *MBL/MBNL* genes as muscleblind and use this term or MBL when discussing this family of genes/proteins. *MBNL1* and *MBNL2* are ubiquitously expressed in adult tissue, however, *MBNL1* is more highly expressed in muscle, and *MBNL2* in brain (4). *MBNL3* is developmentally regulated and is primarily expressed in placental tissue and in muscle satellite cells, playing roles in muscle regeneration and differentiation (5). The three human/mouse *MBNL* paralogs undergo alternative splicing, which affect localization and activity of the proteins. Human *MBNL1* contains 10 exons that can give rise to at least 10 different splice isoforms (6,7). In particular, we focus on the 41 kDa isoform of MBNL1, which contain exons 1–4, 6–8 and 10. *MBNL1* and *2* play a prominent role in the RNA repeat-expansion disease myotonic dystrophy (DM). In this disease, the sequestration of Muscleblind proteins to expanded CUG repeats leads to mis-splicing of several mRNAs, causing several DM symptoms (8). Mammalian MBL is also involved in other RNA metabolic processes such as regulation of gene expression (9,10), mRNA stability (11), alternative 3′ end processing (12), localization (13,14) and microRNA processing (15).

RNA binding by MBL proteins occurs through highly conserved tandem CCCH-type zinc finger (ZnF) domains (16). Many studies have strived to identify RNA motifs recognized by MBNL1 and the mechanism by which it binds to transcripts and regulates splicing. Fly (*Drosophila melanogaster*) MBL and human MBNL1 tend to bind YGCY (Y = C or U) containing RNA motifs (17,18). CLIP-seq and RNA Bind-n-Seq experiments have identified slightly more specific and sub-optimal motifs, including the 4mers GCUU and UGCU, with MBNL1 binding specificity characterized as YGCY + GCUU (14,19).

We analyzed sequence conservation and explored the splicing regulatory capacity of MBL proteins from *Ciona intestinalis* (CiMBL), *Trichoplax adhaerens* (TaMBL) and *Caenorhabditis elegans* (CeMBL), alongside human/mouse MBNL1 and *Drosophila* MBL. Using validated splicing reporter mini-genes in HeLa cells over-expressing MBL protein, we found that these homologs can regulate splicing of specific, exogenous pre-mRNAs. RNAseq experiments accessing global splicing regulation in mouse embryonic fibroblasts (MEFs) stably expressing MBL proteins showed that the homologs also regulate many endogenous targets. MBL proteins from human, *Ciona*, fly, worm and *Trichoplax* are present in the nucleus and cytoplasm. Cytoplasmic localization suggests extra-nuclear activity, which would further extend the functional conservation of non-human MBLs beyond that of splicing regulation. Studying distant MBL proteins may provide insight into ancestral or novel functions that have been conserved from *Trichoplax* to human MBNL proteins.

## MATERIALS AND METHODS

### Cloning, cell lines and splicing reporters

N-terminal HA-tagged DNA constructs encoding HsMBNL1, *Drosophila melanogaster* (DmMBL), CeMBL, CiMBL and TaMBL were cloned into the pCI plasmid (Promega) using standard cloning procedures. HA-tagged HsMBNL1 in pcDNA3 was previously cloned from MBNL1-eGFP (17) obtained from the laboratory of Maury Swanson. HA-DmMBL was cut directly out of a previously cloned construct of HA-DmMBL in pcDNA3 using Kpn1 and Xba1 and inserted into pCI. CeMBL DNA with N-terminal Bam H1 and C-terminal NotI restriction enzyme cut sites was synthesized by GenScript and received in pUC57 cloning vector. KpnI and XbaI restriction enzymes were used to insert Ha-CeMBL into pCI. CiMBL DNA with N-terminal HA-tag and EcoRI cut site and C-terminal Sal1 restriction enzyme cut sites was synthesized by GenScript and received in a pUC57 cloning vector. HA-CiMBL was inserted into pCI using EcoRI and SalI. TaMBL cDNA was generously provided by Dave Anderson (Thornton Lab, University of Oregon, USA). Ha-TaMBL was inserted into pCI using KpnI and XbaI restriction enzymes.

N-terminal GFP-tagged constructs encoding HsMBNL1, DmMBL, CeMBL, CiMBL and TaMBL were cloned into pAC156, a vector containing PiggyBac Transposon (20) sequences flanking an EF1α-driven puromycin cassette, as well as an EF1α promoter present to drive expression of the GFP-tagged construct (gifted by Albert Cheng). The In-fusion cloning system (Clontech) was used according to the manufacturers instructions, to clone the GFP-tagged constructs into pAC156. At 60% confluency, MEFs deficient in MBNL1/2, gifted by Maurice Swanson, were transfected with 2 µg plasmid encoding GFP-Muscleblind using TransIT (Mirus) and 500 ng of PiggyBac transposon to stably introduce GFP-Musclebind into the cells. After 24 h the cells were subjected to puromycin selection (2 µg/ml), allowed to recover for several days and sorted for similar GFP-expression. The cell lines were maintained in puromycin.

**Figure 1.**
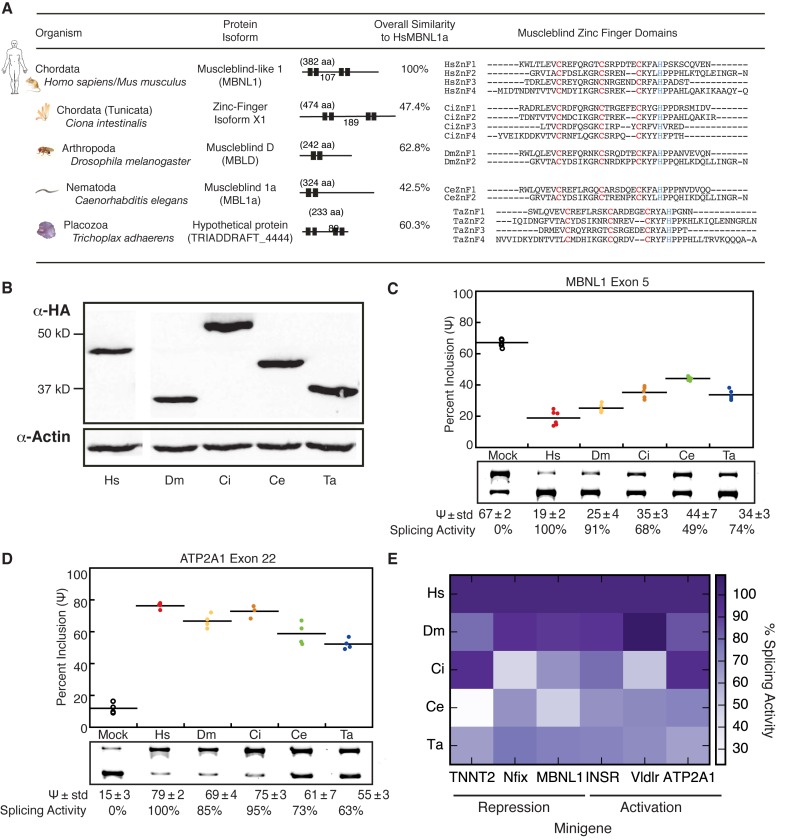
Distant Muscleblind (MBL) homologs regulate splicing in a transcript-dependent manner. (**A**) BLASTX searches, using human MBNL1 (HsMBNL1, identical to mouse MBNL1) as query, identified homologs in *Ciona intestinalis* (CiMBL), *Drosophila melanogaster* (DmMBL), *Caenorhabditis elegans* (CeMBL) and *Trichoplax adhaerens* (TaMBL). DmMBL is overall most similar to HsMBNL1 based on local alignment calculations. Zinc-Finger (ZnF) RNA-binding domains are highly conserved. (**B**) Transfected HeLa cells express similar levels of HA-tagged Muscleblind proteins. (**C**) MBL homologs can activate or repress splicing of a *MBNL1* exon 5 minigene or (**D**) *ATP2A1* exon 22 minigene with varying activity (shown as mean percent spliced in, or Ψ). (**E**) Non-HsMBNL1 splicing activity varies depending on the minigene substrate; darker purple represents higher activity. Splicing activity is displayed relative to HsMBNL1 splicing, where activity is represented as the percentage change in Ψ in the presence of the homolog relative to mock, divided by the percentage change in Ψ in the presence of HsMBNL1 relative to mock (n ≥ 4).

Reporter constructs used for the splicing assay were previously cloned; TNNT2 was gifted from laboratory of Thomas Cooper (21). ATP2A1 (22), *MBNL1* (23) (Figure 1C) and *INSR* were from Nicholas Webster (24) and Nfix, and Vldlr were from Manuel Ares, Jr. (10). The MBNL1 reporter used in Supplementary Figure S4A was previously created by us and described (25).

### Cell culture

HeLa cells were maintained as a cultured monolayer in Dulbecco's modified Eagle's medium (DMEM) + GLUTAMAX media supplemented with 10% fetal bovine serum (FBS) and 10% antibiotic-antimycotic (Gibco, Invitrogen). The cells were kept at a constant temperature of 37°C in a humidified incubator (5% CO_2_). MEF cells (gifted by Maury Swanson) were maintained in DMEM supplemented with 20% FBS, 5% penicillin/streptomycin and 2 µg/ml puromycin at 37°C and 5% CO_2_.

### Western blots

HeLa cells were lysed in RIPA lysis buffer (100 mM Tris pH 7.4, 300 mM NaCl, 10% NP40, 10% Na-deoxycholate, protease inhibitor, 200 mM PMSF, 10% SDS). Protein concentration was quantified using BCA reagent (Thermo Scientific) following manufactures instruction. Total protein lysates (5 µg) were loaded on 12% SDS-Page denaturing gel, electrophoresed for 40 min and transferred via a fast partial wet transfer (200 mA and 100 V for 2 h) to a 0.45 μm pore size nitrocellulose membrane (GE Water & Process Technologies). Following protein transfer, a Ponceau stain (Sigma-Aldrich) was performed in order to ensure proper transfer. TBST was used to wash the nitrocellulose. The blot was blocked for 4 min in 4% milk in TBST prior to administration of the primary antibody. All blots were exposed to primary antibody at a dilution of 1:1000 (antibody: 4% milk in TBST) overnight at 4°C and exposed to secondary antibody at a dilution of 1:2000 for 2 h at room temperature. HA-probe (F7) mouse polyclonal IgG antibody and actin (I-19) rabbit polyclonal IgG (Santa Cruz Biotech) primary antibodies were used.

MEFs grown to 80% confluence were scraped, re-suspended into lysis buffer (20 mM Tris pH 8.0, 200 mM NaCl, 2 mM MgCl_2_, 10% Glycerol, 1% NP40, EDTA-free protease inhibitor tablet (Roche, Mini, EASYpack), PhosSTOP phosphatase inhibitor cocktail tablet (Roche)), incubated 20 min on ice, spun down at 4°C for 10 min at 14 000 rpm. Supernatant was collected and concentration was accessed via Bradford assay. A total of 25 µg protein lysate was loaded on a NuPAGE Bis-Tris Gel (Novex, Life Technologies), electrophoresed and transferred to iBlot2 stacks (ThermoFisher). A ponceau stain confirmed transfer and equal protein loading/sample. Blots were blocked in 5% milk in TBST, exposed over night to primary antibodies (Ms MB1a from the CIND Monoclonal Antibody Resource 1:2000; Rb Anti-GFP (Abcam ab290, GR19763), 1:5000; Rb Anti-βactin (Cell Signaling, #4967S), 1:5000), washed thoroughly and exposed to appropriate secondary antibodies for 2 h at room temperature.

### Reporter splicing assay

HeLa cells were plated in 6-well plates at a density of 1.6–1.8 × 10^6^ cells/well. At 80–90% confluency, cells were co-transfected with 500 ng/well of splicing reporter and MBNL/MBL-encoding or GFP-encoding (mock) plasmid using Lipofectamine 2000 reagent (Invitrogen) in OPTI-MEM reduced serum medium (Gibco, Invitrogen). After 4 h, media was replaced with DMEM+ GLUTAMAX. After 18–24 h, cells were harvested with with TripleE (Gibco, Invitorgen). Experimental procedures follow those previously described (22). RNA was isolated via RNAEasy Kit (Qiagen) according to the manufacturer's instructions and 500 ng of RNA was DNAse-treated (RQ1 DNAse, New England Biolabs). DNAsed RNA [2 µl (100 ng)] was reverse transcribed (RT) with SuperScript II reverse transcriptase (Invitrogen), according to Invitrogen's protocol, except that half the amount of recommended amount of SuperScript II was used. cDNA was PCR amplified for 20–26 cycles using reporter-gene specific primers. PCR products (5 µl) were dyed (Sybr Green DNA loading dye, Invitrogen), resolved on a 6% native acrylamide gel (19:1) and quantified using AlphaImager and associated software (Alpha Innotech). CTG repeat-containing plasmids (DT480 and DT960) were gifted from Tom Cooper and co-transfected with reporter genes.

Percent exon inclusion was calculated by dividing the background-corrected amount of inclusion splice product by the total amount of splice product (background-corrected inclusion splice product + background-corrected exclusion product). Splicing activity relative to human MBNL1 was calculated in the following way: (non-HsMbl – mock inclusion) / (HsMBNL – mock inclusion).

### Immunofluorescence

MEF cell lines expressing GFP-Muscleblind were plated (∼1.25 × 10^5^ cells/well) on collage-coated coverslips (100 µg/µl), incubated overnight (∼18 h) and fixed using 4% paraformaldehyde, 15 min at room temperature. Cells were washed in phosphate buffered saline (PBS), permeabilized in 0.2% Triton-X/PBS at room temperature, 3 min, blocked in 10% bovine serum albumin (BSA)/PBS at 37° for 30 min and exposed to primary antibody, 1:1000 chicken IgY α-GFP (Aves) diluted in PBS + 1% BSA, at 4° overnight. After washing in PBS, the secondary antibody, α-chicken 488 at 1:400 and 594 phalloidin (LifeTechnologies) at 1:400 diluted in PBS + 1% BSA, were incubated on the coverslips at 37°C for 1 h. Coverslips were washed in PBS and subject to Hoechst stain at room temperature for 10 min before final PBS washes and mounting onto microscopic slides. All cells in Figure 3 and Supplementary Figure S2A were imaged on an Applied Precision DeltaVision Microscope at 60X magnification, optical sections were deconvoluted using the associated software, and processed using ImageJ. Adjusted intensity projections were generated from the average of three z stacks, centered around the nucleus. HeLa cells, transfected with HA-Muscleblind, were processed and imaged similarly except that they were fixed after a 2 h incubation; HA-probe (F7) mouse polyclonal IgG primary antibody (1:1000) was used, and images represent a single z stack. Cells in Supplementary Figures S2B and S4B were imaged on a Zeiss LSM880 at 63x magnification using confocal microscopy. Maximum Z-projections are shown. FISH against CUG repeats was performed as previously described (26), using a (CAG)_10_ probe conjugated to CalFluor 610 (Biosearch Technologies).

### RNAseq

Total RNA was isolated from GFP-Muscleblind expressing MEF cells using Direct-zol RNA columns (Zymo Research) according to manufacturer's instructions. cDNA libraries were generated starting with 1 µg RNA. Briefly, RNA was fragmented, depleted of ribosomal RNA using Ribo-Zero-Gold kit (Epicentre), and reverse transcribed followed by end-repair, adenylation and adapter ligation. Unique barcodes were used for each library to allow for multiplexing all samples in a single lane (80 + 80 bases, paired-end, NextSeq). STAR (27) was used to align reads to the mouse mm9 genome, and MISO (28) was used to quantify splicing. All RNAseq data have been deposited in GEO under accession number GSE79095.

### Motif analysis

The frequency of GCTT, CGCT, TGCT or GCGC (MBNL binding) motifs was compared to the frequency of control 4-mers with the same A+T and CpG content in the skipped exon and 250 bp of introns downstream of the upstream constitutive exon, upstream of the skipped exon, downstream of the skipped exon and upstream of the downstream constitutive exon. Skipped exons were grouped into those activated by MBNL homologs (BF > 5, ΔΨ > 0.1, Z > 1.5 in WT versus DKO), those repressed by MBNL homologs (BF > 5, ΔΨ < −0.1, Z > 1.5 in WT versus DKO), or those not affected by MBNL homologs. The log_2_(enrichment of MBNL binding motifs relative to controls in activated exons/enrichment of MBNL binding motifs relative to controls in unaffected exons) was plotted in heatmap form. A similar metric was plotted for repressed exons. Genome sequence for mouse mm9 was downloaded from UCSC genome browser.

### Gene ontology analysis

For each species, multiple alignments were used to analyze the branch length across which MBL/MBNL binding motifs are conserved in the 150 nucleotides flanking all internal exons. Multiple alignments and branch lengths for human, mouse, fly and worm were downloaded from UCSC genome browser; multiple alignments for *Ciona* were downloaded from the Joint Genome Institute. For human, Refseq gene annotations were used; multiple alignments included hg38, panTro4, rheMac3, mm10, rn5, canFam3 and monDom5. For mouse, Refseq gene annotations were used; multiple alignments included mm9, rn4, canFam2, bosTau3, monDom4, galGal3, xenTro2, danRer5 and tetNig1. For fly, Refseq gene annotations were used; multiple alignments included dm6, droSim1, droYak3, droAna3, droPse3, droVir3, droMoj3, anoGam1 and apiMel4. For worm, Wormbase gene annotations were used; multiple alignments included ce10, cb4, caeRem4, caeSp111, caePb3, caeJap4 and caeAng1. For *Ciona*, Ensembl gene annotations were used; a pairwise alignment of *Ciona intestinalis* and *Ciona savignyi* was downloaded from JGI (http://pipeline.lbl.gov/data/Cioin2_cioSav2/). For each intronic region, the branch length across which 4-mer motifs were conserved was recorded. The mean branch length of GCTT, CGCT, TGCT or GCGC (MBL/MBNL binding) motifs, less the mean branch length of control motifs with the same A+T and CpG content, yielded δ_intron_. The largest δ_intron_ for all introns in each gene was kept as δ_gene_. Then, for each Gene Ontology category, the distribution of δ_gene_ for genes within the category was compared to the distribution of δ_gene_ for all genes outside the category, using a ranksum test. *P*-values were Benjamini–Hochberg corrected, and *P*-values were converted to 1 for those categories with lower mean δ_gene_ as compared to controls.

## RESULTS

### Muscleblind homologs regulate splicing of mammalian mini-gene reporters

To gain insight into conservation of function of the MBL family of RNA binding proteins, we studied MBL proteins from five diverse organisms including *Homo sapiens/mus musculus* (protein coding sequences are identical); *Ciona intestinalis*, a primitive chordate; *Drosophila melanogaster* and *Caenorhabditis elegans*, two commonly used model organisms; and *Trichoplax adhaerens*, a basal metazoan. Using the human/mouse 41 kD isoform of *MBNL1* sequence as a query, we performed BLAST searches to identify homologs in these organisms. The 41 kD isoform of *MBNL1* contains the linker region between both pairs of zinc fingers (exon 3), lacks exon 5, which can control nuclear/cytoplasmic localization, and lacks exon 7, which has been proposed to contribute to MBNL1 dimerization (6). This isoform has been extensively studied and exhibits both nuclear and cytoplasmic distribution (29). *Zinc-finger X1* in *Ciona, Muscleblind D* in *Drosophila, Muscleblind-1a* in *Caenorhabditis* and a hypothetical protein in *Trichoplax* were identified as the highest scoring homologs. For clarity, we refer to these proteins as HsMBNL1, CiMBL, DmMBL, CeMBL and TaMBL for human, *Ciona, Drosophila, Caenorhabditis* and *Trichoplax* Muscleblind proteins, respectively. To quantify the extent of overall protein homology, Smith–Waterman alignments comparing HsMBNL1 to each homolog, and the associated similarity scores, which reflect aligned residues with similar properties, were calculated (Figure 1A). DmMBL showed the highest overall similarity score of 62.8%, followed by TaMBL, CiMBL and CeMBL.

The Muscleblind family contains CCCH-type ZnF RNA-binding domains, normally occurring as a tandem pair. Our multi-species alignment shows that the ZnF domains are highly similar and are the most conserved regions of these proteins (Figure 1A, far right), despite the fact that the number of ZnFs, internal spacing between the conserved cysteine and histidine residues, and spacing between tandem ZnF domains vary among each homolog. Some regions outside of the ZnF domains are also conserved and have been previously shown to be important for MBL function. The motifs RD/KWL, or LEV box, and KxQL/NGR, which closely flank the first ZnF pair, are involved in nuclear localization for human MBNL1 (3,7), and may be present in DmMBL, CeMBL and TaMBL (Supplementary Figure S1). The linker region between tandem ZnF pairs exhibit the next highest density of conserved residues outside of the ZnFs. Mutation and truncation analysis in this linker region has demonstrated its importance for human MBNL splicing activity (6,30). Finally, numerous proline residues are conserved, some of which lie in the linker region and have been previously shown to interact with Src family kinases and alter their activity (31). The overall similarity between the MBL proteins in our study, particularly within the RNA-binding domains, suggests conserved RNA binding and provided the rationale for investigation of conserved splicing regulation by these homologous proteins.

To initially explore splicing of the MBL homologs, we conducted cell-based splicing assays using mini-gene reporters. Transfection of HA-tagged expression vectors encoding each homolog yielded similar, high expression levels in HeLa cells, as assessed by Western blot (Figure 1B). The homologs were also observed in both nuclear and cytoplasmic compartments of transfected HeLa cells (Supplementary Figure S2A). Splicing reporter mini-genes, including human cardiac troponin T type 2 (*TNNT2*) exon 5 (17), mouse nuclear factor I/X (*Nfix*) exon 8 (10), human *MBNL1* exon 5 (23), human sarcoplasmic/endoplasmic reticulum Ca^2+^-ATPase 1 exon 22 (*ATP2A1*) (22), mouse very-low-density lipoprotein receptor (*Vldlr*) exon 16^10^, and human insulin receptor exon 11 (*INSR*) (32) were co-transfected with MBL/MBNL homolog or GFP (control). Inclusion levels of each alternative exon was quantitated by RT-PCR, and expressed as percent spliced in (Ψ) and also splicing activity as a percentage of activity relative to HsMBNL1. Across all mini-gene reporters tested, we observed the same direction of splicing regulation (either activation or repression) for all homologs, with varying degrees of regulation relative to HsMBNL1. For example, expression of HsMBNL1 represses inclusion of MBNL1 exon 5 from 67% to 19%; all MBL homologs exhibited similar repressive activity, but at varying degrees, with CeMBL exhibiting the weakest activity (Ψ = 44%) (Figure 1C). Similarly, all MBL homologs could enhance inclusion of *ATP2A1* exon 22, raising Ψ from 15% to between 55–79% (Figure 1D).

Homologs that most strongly repressed MBNL1 exon 5 were not necessarily those that most strongly enhanced ATP2A1 exon 22. Assessment of splicing activity using additional reporters revealed that potency of MBL/MBNL splicing activity depends on the specific transcript (Figure 1E, Supplementary Figure S3). These differences in activity may be due to differences in *cis*-element architecture of the reporters, and also differences in the MBL/MBNL activity due to sequence variation between homologs. Among the reporters tested, DmMBL exhibited the most potent splicing activity relative to HsMBNL1, CeMBL was the poorest regulator for two of the reporters and TaMBL exhibited 60–80% activity, regardless of reporter. These results are consistent with previous data showing that HsMBNL1 and DmMBL act as both activators and repressors of alternative splicing; here, we observe that CeMBL, CiMBL and TaMBL also exhibit these activities.

### MBNL1/2 null cells reconstituted with MBL homologs were generated to test global splicing regulation

**Figure 2.**
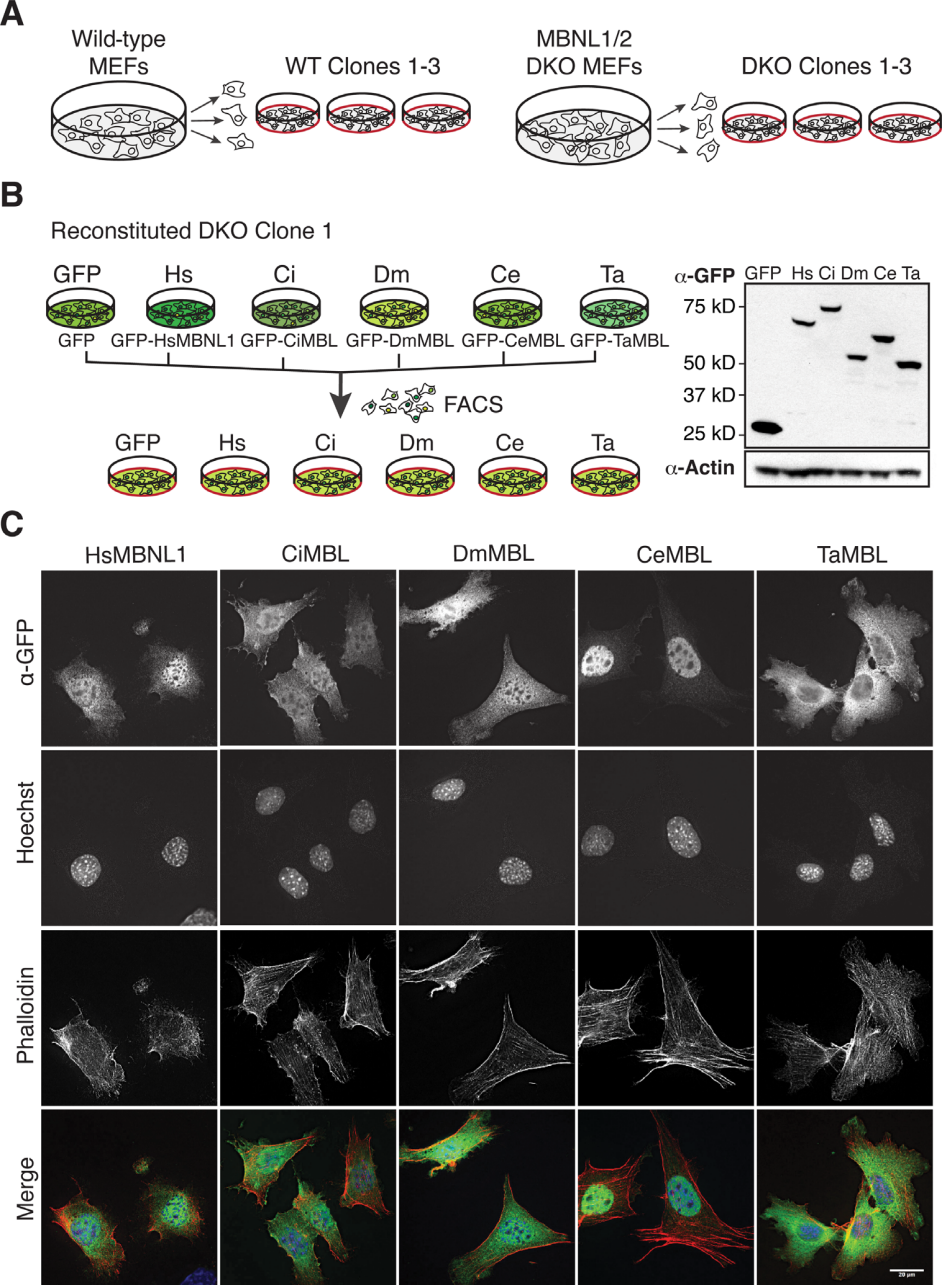
RNAseq libraries were generated from WT, MBNL1/2 knockout (DKO) and Muscleblind-reconstituted mouse embryonic fibroblasts (MEFs). (**A** and **B**) MBNL1/2 double knockout MEFs (DKO) were reconstituted with GFP or GFP-tagged Muscleblind proteins from a single DKO clone, and sorted to select for similar GFP expression. RNAseq libraries were prepared from three WT clones, three DKO clones and the sorted, reconstituted clones (plates with red outlines). Each clone expresses similar levels of MBL/MBNL homolog, as assessed by Western blot. (**C**) Immunofluorescent staining of each reconstituted line shows MBL proteins in both nucleus and cytoplasm. Anti-GFP (MBL/MBNL1), green; Hoechst (nuclear stain), blue; phalloidin 594 (F-actin), red; scale bar, 20 μm.

To assess the ability of the MBL homologs to regulate endogenous transcripts across the transcriptome, independent of potential over-expression artifacts, we established stable cell lines expressing each homolog. First, to identify endogenous targets of mouse MBNL proteins, we performed RNAseq on 3 independent wild type MEF clones and 3 independent *MBNL1/2* knockout MEF clones (double knockout, or DKO, Figure 2A). We then selected one of the DKO clones and stably integrated an expression cassette containing each N-terminal GFP-tagged homolog, driven by the EF1α promoter, along with a puromycin selection cassette. Following puromycin selection, we used fluorescent-activated cell sorting to select cells that express similar levels of GFP-tagged MBL homolog. RNAseq libraries were generated from the five cell lines and an additional line expressing GFP alone (Figure 2B). Expression levels of GFP-tagged homologs were comparable across all lines, as assessed by Western blot (Figure 2B).

We determined the subcellular localization of each homolog in our cell lines using immunofluorescence against GFP (Figure 2C). All homologs showed some nuclear and cytoplasmic distribution, consistent with previous observations of the 41 kD isoform of HsMBNL1, of DmMBL, and CeMBL (6,33,34). The control, GFP-expressing cell line also showed localization to both nuclear and cytoplasmic compartments (Supplementary Figure S2B). Differences in nuclear-cytoplasmic localization may be due to differences in protein sequence among the homologs, cell type, cell state and/or extracellular signals, but presence in the nucleus is consistent with a conserved function in regulation of splicing.

### Endogenous exons are regulated by MBL homologs in reconstituted DKO cells

**Figure 3.**
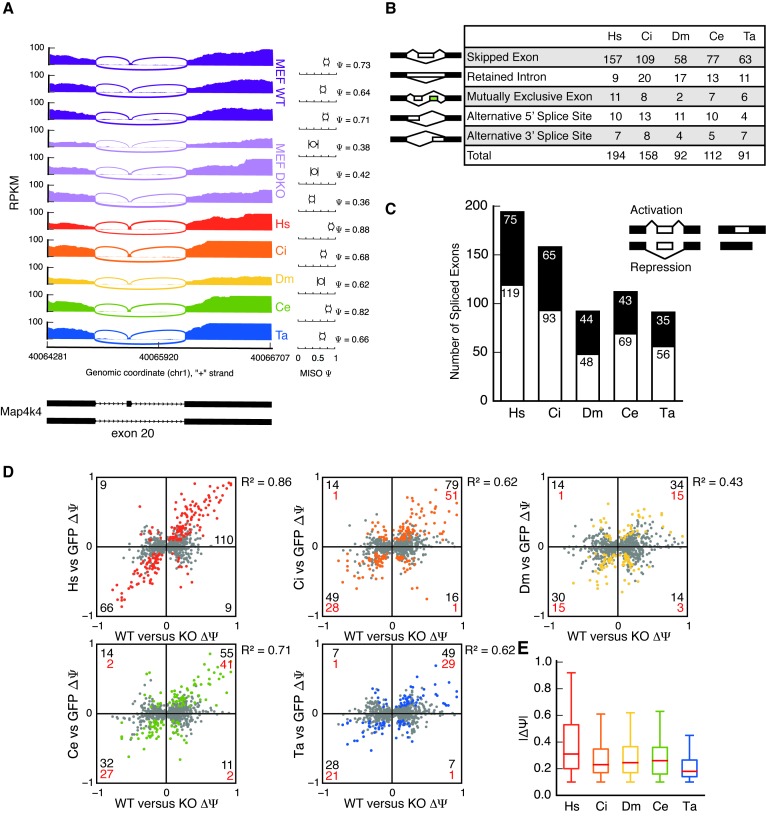
Distant MBL homologs regulate splicing of many endogenous exons in a manner similar to human MBNL1. (**A**) RNAseq read coverage across *Map4k4* exon 20 shows an example of alternative splicing regulation in WT, DKO and MBL-reconstituted MEFs. MISO Ψ values and 95% CIs are shown at right. (**B**) All types of alternative splicing are regulated by MBL homologs. (**C**) Number of spliced exons activated (white bar) or repressed (black bar) by MBL homologs. Hs and Ce show significantly more splicing activation than repression. (**D**) Reconstitution of MBL homologs rescues splicing in MBNL1/2 DKO MEFs. Exons significantly regulated in WT MEFs: gray points; exons significantly regulated in reconstituted cell lines: red (Hs), orange (Ci), yellow (Dm), green (Ce), blue (Ta) points. Black numbers in quadrants show the number of exons significantly regulated in reconstituted cells. Red numbers show the number of exons significantly regulated by both HsMBNL1 and associated MBL homolog. (**E**) Boxplot of absolute splicing changes, |ΔΨ|, for colored points in (D) shows that HsMBNL1 exhibits the strongest splicing regulation, whereas TaMBL exhibits the weakest.

RNAseq reads were mapped by STAR (27) to the genome and splice junctions, yielding 76M, 51M, 65M, 51M, 38M and 70M uniquely mapping reads from GFP, Hs, Ci, Dm, Ce and Ta-reconstituted lines, respectively. Psi values were quantitated using MISO (28) across all alternative splicing events. Clear examples of MBNL-regulated events that could also be regulated by each of the homologs were observed. For example, *Map4k4* exon 20 is included from 64–73% in WT MEFs, but at 36–42% in DKO MEFs; homolog expression was sufficient to enhance inclusion to 62–88%, depending on the homolog (Figure 3A). We further analyzed alternative splicing events across the transcriptome, focusing on events that were MBNL-dependent (those that exhibit a monotonicity Z-score ≥ 1.5 between WT and DKO MEFs, Supplementary Table S1) and could be rescued by any given homolog (MISO Bayes Factor ≥ 5 and |ΔΨ| ≥ 0.1, Supplementary Table S2). We identified 194, 158, 92, 112 and 91 exons significantly regulated in Hs, Ci, Dm, Ce and Ta-MBL reconstituted cells, respectively (Figure 3B, Supplementary Table S2). All major classes of splicing event types were represented, including skipped/cassette exons, retained introns, mutually exclusive exons and alternative 5′ or 3′ splice sites. Both splicing activation and repression activities were observed for each homolog, with a slight bias toward exon inclusion for Hs and Ce (binomial *P*-values < 0.01) (Figure 3C).

Across the transcriptome, significantly regulated splicing changes in cells lacking MBNL1/2 strongly correlated with splicing changes in DKO cells re-constituted with HsMBNL1 (R^2^ = 0.86, Figure 3D). Other homologs also showed positive correlations, with R^2^ values ranging from 0.43 (Dm) to 0.71 (Ce). In general, the direction of splicing regulation (activation or repression) by MBL homologs was consistent with regulation by endogenous MBNLs; the number of events regulated by one or more homologs is shown in Supplementary Figure S3B. To compare the extent to which each homolog regulates splicing, we plotted the magnitude of splicing regulation for each ortholog (|ΔΨ|) relative to control (GFP-expressing) cells. HsMBNL1 and TaMBL exhibited the most and least dramatic regulation, respectively, as assessed by median |ΔΨ| relative to control (GFP-expressing) cells (Figure 3E).

**Figure 4.**
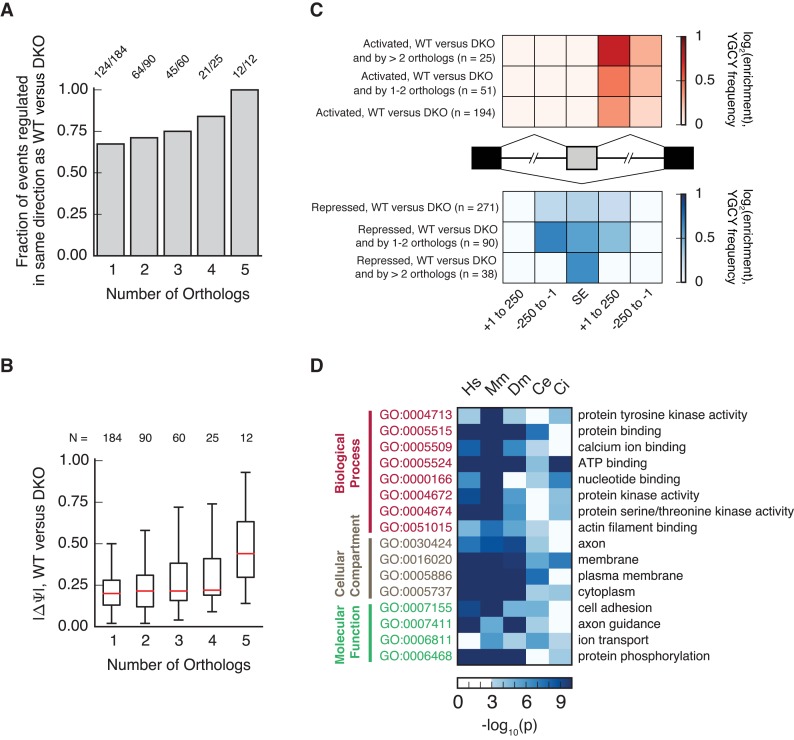
Splicing events regulated by multiple MBL/MBNL homologs are enriched for properties typical of MBL/MBNL targets. (**A**) The fraction of events in which 1–5 orthologs regulate each splicing event (BF > 5, |ΔΨ| > 0.1) in the same direction as observed in WT versus DKO is shown in bars. (**B**) Absolute change in splicing (|ΔΨ|) is plotted in boxplots for events that are regulated by 1–5 orthologs, with medians in red. (**C**) YGCY enrichment is shown for regions adjacent to exons regulated by MBNL orthologs in a heatmap for activated (top) or repressed (bottom) events. One row is shown for events regulated between WT and DKO cells, those also regulated by 1–2 orthologs, and those regulated by 3 or more orthologs. (**D**) Enrichment of Gene Ontology categories is shown for genes containing introns with highly conserved MBL/MBNL binding sites, where –log_10_(p) of enrichment is shown as blue shading in a heatmap. Only categories significantly enriched (*P* < 0.01, Benjamini–Hochberg corrected) in at least four organisms are shown.

Splicing events that were significantly regulated by a greater number of homologs tended to be regulated in a direction consistent with that observed when comparing WT to DKO MEFs (Figure 4A). In addition, the magnitude of splicing regulation conferred by MBNL, as assessed when comparing WT versus DKO MEFs, was larger for those events regulated by a greater number of homologs relative to those regulated by fewer homologs (Figure 4B, Supplementary Table S2). As has been previously observed, YGCY motifs are enriched in the introns downstream of exons activated by MBNL, and in the upstream introns and exons repressed by MBNL, relative to control exons, as assessed when comparing WT to DKO MEFs (Figure 4C). The magnitude of this enrichment was slightly increased among exons regulated by more than two homologs (Figure 4C).

### MBNL homologs can rescue CUG repeat-dependent mis-splicing, and colocalize with expanded CUG repeats

Given the ability of each homolog to regulate exons in a manner similar to human/mouse MBNL, we tested their abilities to rescue CUG repeat-mediated aberrant splicing of MBNL1 exon 5, TNNT2 exon 5, Nfix exon 8 and ATP2A1 exon 22 in the context of a minigene reporter. Co-transfection of each homolog in HeLa cells could partially rescue mis-splicing (Supplementary Figure S4A), suggesting that each homolog may compete for binding to CUG repeats to release endogenous MBNLs, through recognizing a binding site similar to that recognized by human/mouse MBNLs. In further support of this hypothesis, we also tested the ability of each homolog to bind to expanded CUG repeats. Transfection of a plasmid encoding 480 CTG repeats into the reconstituted MEFs yielded GFP foci in the nucleus of each cell line (Supplementary Figure S4B), although fly MBL did not yield foci as strong as the other homologs. This fly isoform has been previously demonstrated to bind CUG repeats more weakly than the MBNL binding site found in intron 4 of cardiac troponin T (18).

### Enrichment of MBNL binding motifs in introns of specific Gene Ontology categories across evolution

To gain insight into properties of MBL/MBNL target exons throughout evolution, we analyzed exons in species with reasonably comprehensive gene ontology annotations (*Trichoplax* was omitted from this analysis). We used motif conservation to predict MBL/MBNL target exons, and asked whether particular gene ontology categories were enriched among putative MBL/MBNL target genes. We first computed branch length conservation for top MBL/MBNL target 4-mers, as previously assessed by RNA BindNSeq (19), within introns −150 to −1 upstream of each exon and +1 to +150 downstream of each exon. Branch length conservation was assessed using multiple alignments of human introns relative to 6 vertebrate species, mouse versus 8 vertebrates, *D. melanogaster* versus 8 insects, C*. elegans* versus 5 worms and *C. intestinalis* versus C*. savignyi*. We asked whether the mean branch length of MBNL motifs was higher than expected, relative to control motifs, in genes within each gene ontology category. Interestingly, we found that gene ontology categories containing the most highly conserved MBL/MBNL motifs were similar across broad evolutionary time (Figure 4D). For example, ‘calcium ion binding’, ‘ATP binding’ and ‘actin filament binding’ were enriched across at least 4 of the 5 analyses, as well as ‘membrane’, ‘ion transport’ and ‘cell adhesion’. While specific target exons of MBL/MBNL proteins have not necessarily been conserved across such large evolutionary distances, a role for MBL/MBNL in mediating the splicing of genes involved in particular biological functions may be conserved across millions of years of evolutionary time.

## DISCUSSION

### Diverse Muscleblind homologs regulate alternative splicing of mammalian transcripts

Splicing reporter assays in HeLa cells and RNAseq analysis of endogenous transcripts in MEFs reconstituted with MBL proteins from fly, worm, *Ciona* or *Trichoplax* showed conserved alternative splicing regulation that positively correlated with splicing activity observed with the mammalian MBNL proteins (Figures 1 and 3). These observations indicate a strongly conserved role for Muscleblind proteins as regulators of alternative splicing across all metazoans. A multi-species alignment between the homologs revealed that the CCCH-type ZnF–RNA binding regions are the most conserved regions between species (Figure 1A and Supplementary Figure S1). A lack of conservation existing outside of the RNA binding domains suggests that these proteins regulate splicing primarily via interactions between ZnF domains and YGCY binding sites found in RNAs of MBL/MBNL-regulated events.

Differences observed in splicing regulation by the homologs may be attributable to differences in the number of ZnFs, differences in ZnF binding affinity or specificity, and spacing between tandem ZnF domains. These ZnF properties have been previously shown to affect HsMBNL1–RNA interaction and splicing regulation (35). While we did not observe an obvious correlation between homolog ZnF number and the extent of splicing regulation, it is plausible that some substrates are more strongly regulated by MBL homologs containing a greater number of ZnFs. The *Drosophila* homolog we studied contains 2 ZnFs, but homologs with 4 ZnFs have also been described (36); it would be interesting to compare their activities.

### Context-dependent splicing activity of Muscleblind is conserved across metazoans

Our results are consistent with previous studies that have shown that other alternative splicing factors have maintained their RNA regulatory maps (binding motifs upstream of exons and within exons correlates with repression and motifs present downstream of exons correlates with activation). The best studied is *Nova*, whose RNA binding protein domains are 94% identical across vertebrates (37). *Nova* homologs from Sea anemone, fly and lancelet can regulate several splicing events across different species (38), and *Pasilla* (*Drosophila* Nova homolog) globally regulates splicing using an RNA regulatory map similar to mouse (39). However, while *Nova* binding site clusters are moderately conserved to chicken and zebrafish (37), few exons have been discovered to be conserved between mammals and fly (39). The 12 splicing events regulated by all MBL homologs are notable for the large ΔΨ for these events (Figure 4B), which is twice the mean ΔΨ for events regulated by four or fewer homologs. A possible explanation for why these events are regulated by all homologs is that these exons contain multiple and redundant MBL/MBNL binding sites that facilitate robust binding and splicing regulation. Enrichment of YGCY motifs downstream of activated exons and within repressed exons regulated by more than two homologs is consistent with this model (Figure 4C).

### Muscleblind homologs localize to the nucleus and cytoplasm

Nuclear localization of MBL/MBNL homologs is consistent with a conserved nuclear function and splicing activity (Figure 2 and Supplementary Figure S2). Varying levels of nuclear/cytoplasmic localization across the homologs could partially or fully explain the differences in splicing activity. For example, CeMBL is located primarily in the nucleus (Figure 2) and exhibits the strongest correlation (R^2^ = 0.71) to WT cells after HsMBNL1 (Figure 3). These homologs may also exhibit other functions in the cytoplasm; the MBL family has been implicated in regulating mRNA export/localization, mRNA decay and alternative 3′ end processing (11–14). It will be of interest to determine whether extra-nuclear functions of MBL/MBNLs are also conserved across evolutionary time.

### Genes with conserved Muscleblind binding motifs may be involved in similar biological pathways

We predicted splicing targets of MBL homologs in human, mouse, worm, fly and *Ciona*, by identifying genes containing highly conserved MBL/MBNL motifs. Surprisingly, these analyses revealed that even though specific target exons may not be conserved throughout metazoan evolution, MBL proteins are predicted to regulate the splicing of genes involved in similar biological pathways, such as cell adhesion, axon guidance and ion transport. While our pathway analyses extended across human, mouse, fly, worm and *Ciona*, we were not able to assess biological pathways in *Trichoplax* that may be regulated by MBL/MBNLs, due to a lack of robust annotations. Given that *Trichoplax* is a very simple organism, with only six described cell types and no evident sensory, muscle or nerve cells (40), the function of *Trichoplax* MBL in its native context may be interesting to investigate, and potentially reveal some of the most ancient functions of the MBL family.

## Supplementary Material

SUPPLEMENTARY DATA
