# Supplementary material for: Conservation of context-dependent splicing activity in distant Muscleblind homologs
Source: Nucleic Acids Res. 2016 Aug 23;44(17):8352–62. doi: 10.1093/nar/gkw735 (PMC5041496; doi:10.1093/nar/gkw735)
Supplement: SUPPLEMENTARY DATA [file supp_44_17_8352__index.html]

Conservation of context-dependent splicing activity in distant Muscleblind homologs — Conservation of context-dependent splicing activity in distant Muscleblind homologs — SUPPLEMENTARY DATA 

# Conservation of context-dependent splicing activity in distant Muscleblind homologs

## SUPPLEMENTARY DATA

- SUPPLEMENTARY DATA
- SUPPLEMENTARY DATA
- SUPPLEMENTARY DATA
- SUPPLEMENTARY DATA
- SUPPLEMENTARY DATA
- SUPPLEMENTARY DATA
- SUPPLEMENTARY DATA
- SUPPLEMENTARY DATA
