# Supplementary figures and images for: Conservation of context-dependent splicing activity in distant Muscleblind homologs
Source: Nucleic Acids Res. 2016 Aug 23;44(17):8352–62. doi: 10.1093/nar/gkw735 (PMC5041496; doi:10.1093/nar/gkw735)

### Supplementary Figure 1

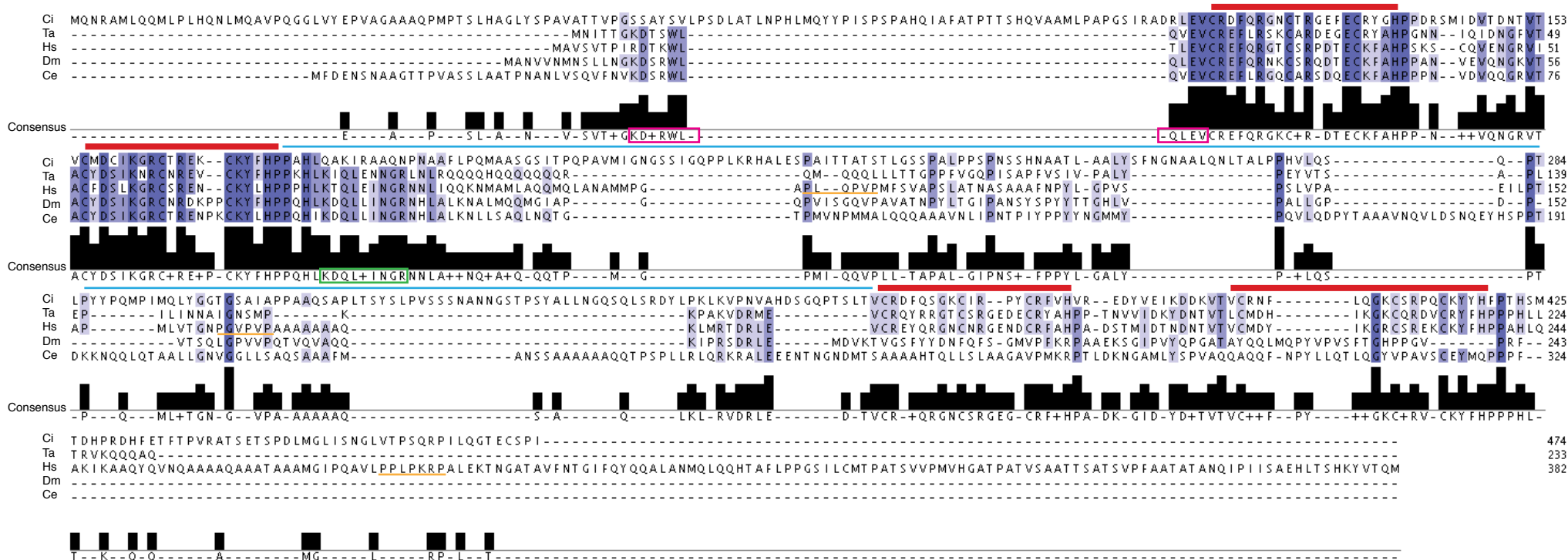

Supplement: SUPPLEMENTARY DATA [file supp_gkw735_nar-01043-a-2016-File006.pdf]

Supplementary Figure 2

A

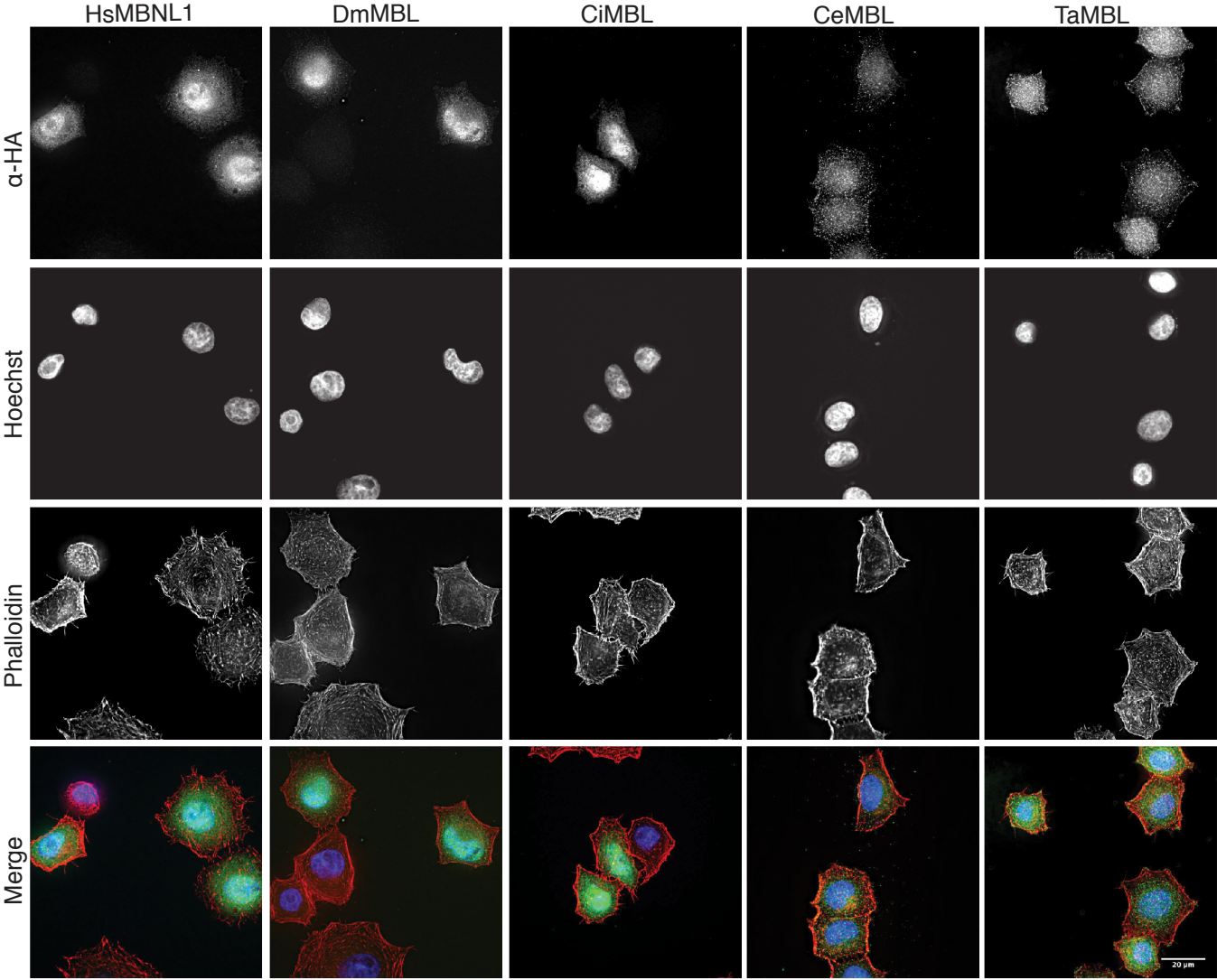

B

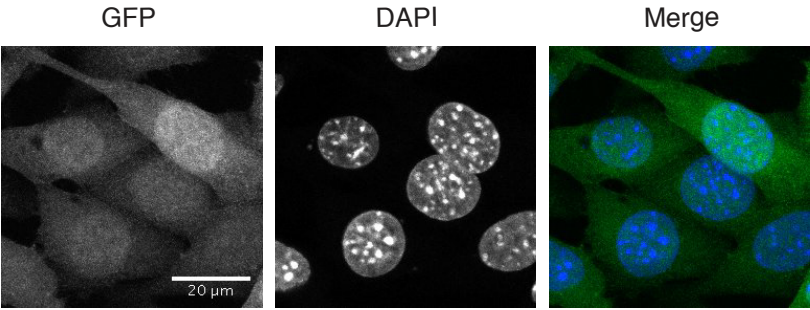

Supplement: SUPPLEMENTARY DATA [file supp_gkw735_nar-01043-a-2016-File007.pdf]

**A**

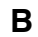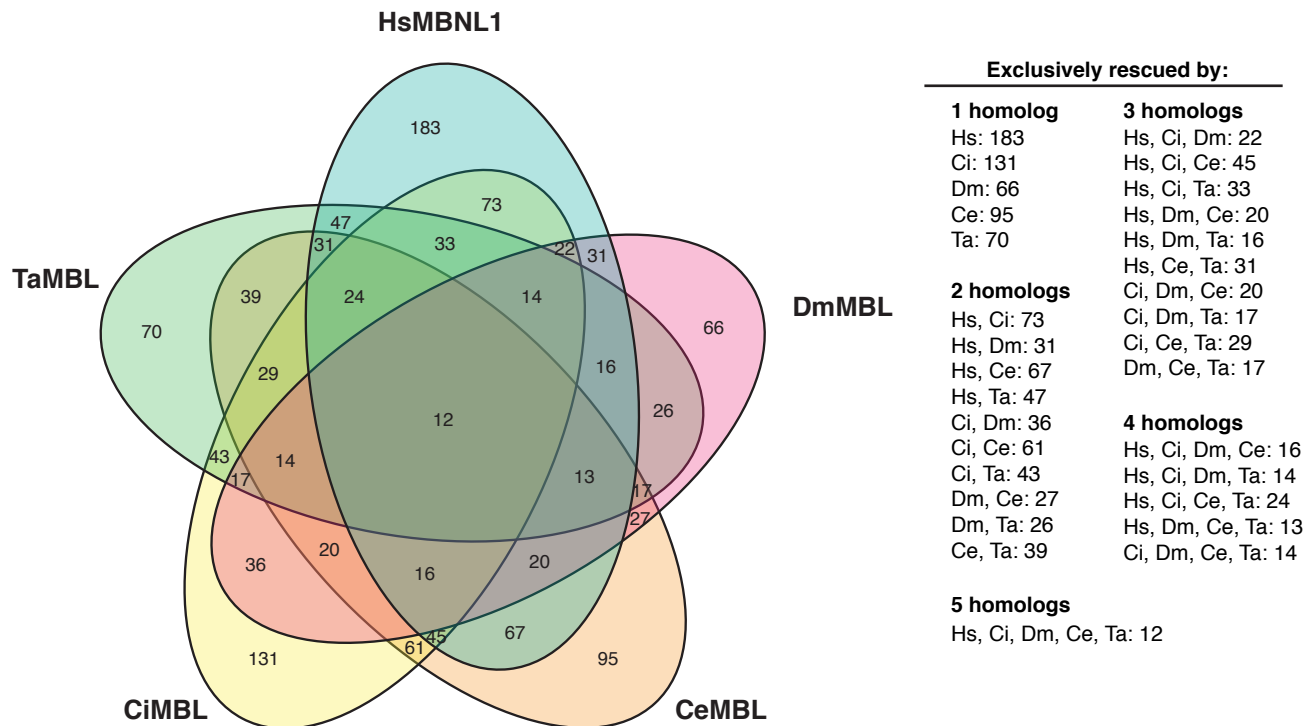

Supplement: SUPPLEMENTARY DATA [file supp_gkw735_nar-01043-a-2016-File008.pdf]

Supplementary Figure 4

A

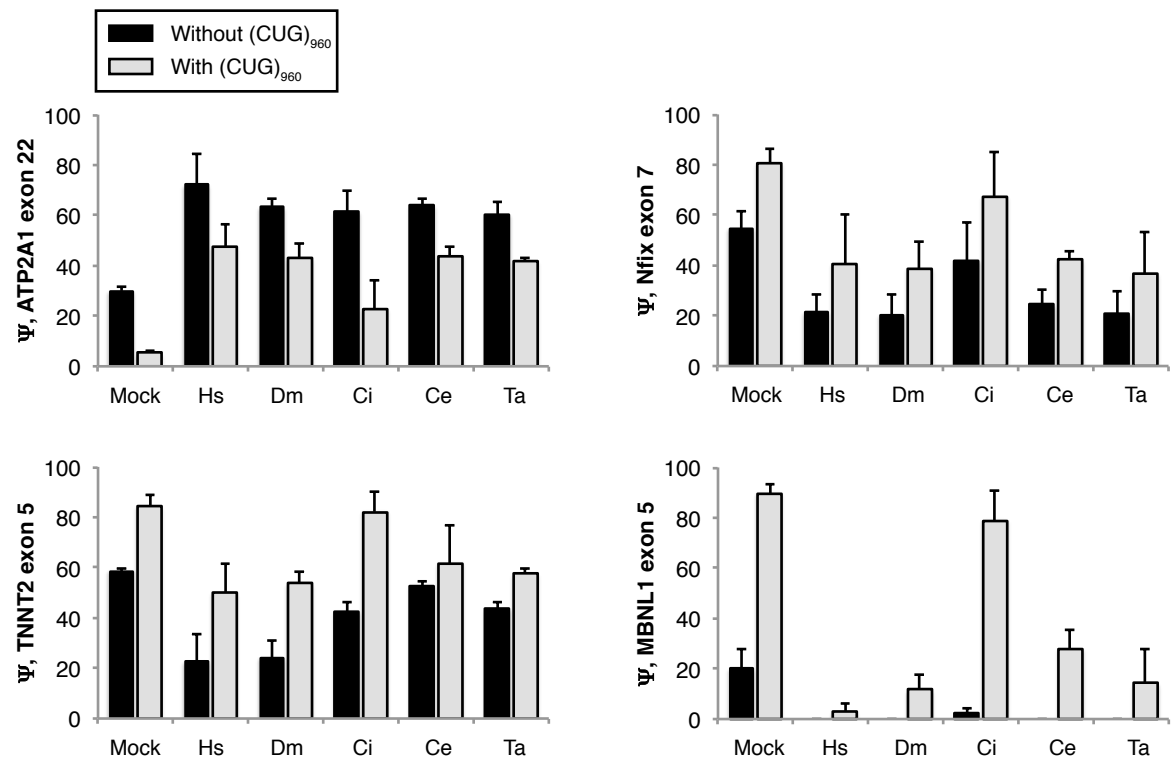

B

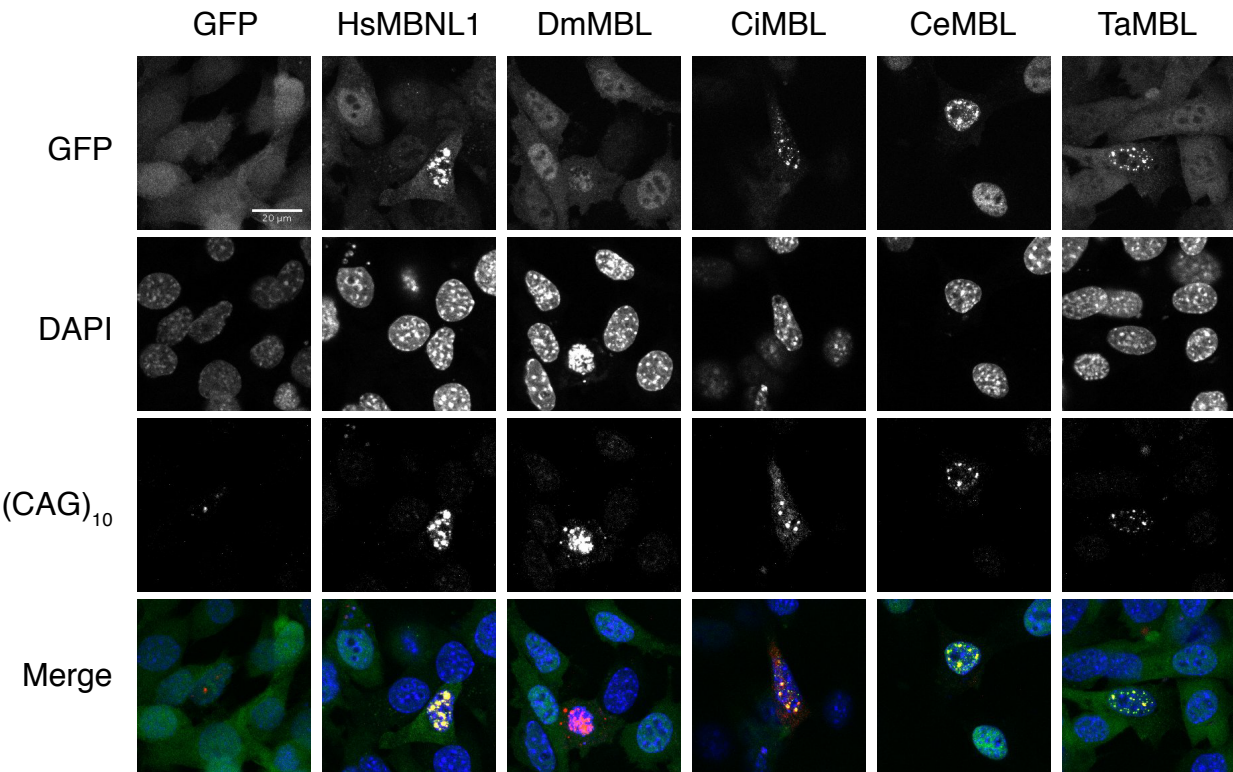

Supplement: SUPPLEMENTARY DATA [file supp_gkw735_nar-01043-a-2016-File009.pdf]
